# A Retrospective Study of Degenerative Cervical Myelopathy and the Surgical Management Within Northern Ireland

**DOI:** 10.7759/cureus.49513

**Published:** 2023-11-27

**Authors:** Laura M Saunders, Hushil S Sandhu, Lorcán McBride, Vindhya S Maniarasu, Samantha Taylor, Rakesh Dhokia

**Affiliations:** 1 School of Medicine, Dentistry and Biomedical Sciences, Queen's University Belfast, Belfast, GBR; 2 Department of Trauma and Orthopaedics, Royal Victoria Hospital, Belfast, GBR; 3 Department of Medicine, Royal Infirmary Hospital, Edinburgh, GBR

**Keywords:** spinal cord compression injury, length of stay, retrospective, northern ireland, orthopaedic, degenerative cervical myelopathy

## Abstract

Introduction: Degenerative cervical myelopathy (DCM) is a condition of growing concern due to its increasing incidence among the ageing population. It involves age-associated pathological changes of the cervical spine that can result in spinal cord compression. This can lead to deficits in motor and sensory function of the upper and lower limbs, issues with balance and dexterity, as well as bladder and bowel disturbance. Patients can be categorised as having mild, moderate, or severe degenerative cervical myelopathy depending on their modified Japanese Orthopaedic Association (mJOA) score. This condition is generally managed surgically; however, patients with mild degenerative cervical myelopathy may be offered or opt for non-surgical treatment initially.

Aims: The main aim of this study is to evaluate the surgical management of patients with DCM and to ascertain the degree of mJOA improvement from pre-surgery and one-year post-surgery follow-up. The second aim of the study is to explore the demographics within Northern Ireland who are diagnosed with DCM and who undergo surgery. This information could allow for better planning of services in the future for this patient cohort.

Methods: This is a retrospective review of the surgical management of degenerative cervical myelopathy within the Regional Spinal Orthopaedic Unit in Northern Ireland over three years with one-year follow-up. The data was retrospectively collected from the Fracture Outcome Research Database. A total of 102 patients (10:7, male:female) with DCM were retrospectively evaluated. Exclusion criteria included all patients diagnosed with spinal tumour, fracture, central cord syndrome, and dislocation. Two patients were removed due to incorrect coding of DCM diagnosis and were not included. Key variables assessed were gender, age, symptoms, type of surgery, complications, and MRC score and mJOA score pre-surgery, 48 hours, six months, and one year post surgery. The choice of surgery was guided by the maximal angle of compression, the number of vertebral levels involved, patient comorbidities, and anesthetic risk.

Results: The sample consisted of 60 men (58.82%) and 42 women (41.17%) with an average age of 57.17 ± 12.13 years ranging from 27 to 83 years old. Statistical analysis was conducted to explore the effect of time before and after surgery up to one year on the mJOA score. There was a significant difference in mJOA score pre-surgery and at six months and one year post surgery (R = 0.579053, p <0.001). Of the patients, 61.8% with a length of stay greater than three days and 71.4% of patients with a length of stay greater than seven days had a posterior approach surgery. A multiple linear regression analysis revealed that the mJOA score pre-surgery and the presence of complications significantly predicted the length of stay post-surgery (β -1.044, p = .011 and β -5.791, p = .028).

Conclusion: The first key finding of this study is that the mJOA score tends to improve after surgery for the majority of patients, particularly at six months, which is consistent with the literature. The second key finding is that anterior approach surgery is associated with a lower rate of complications and shorter post-surgery length of stay in hospital compared to posterior approach surgery. The third key finding is that the pre-surgery mJOA score and the presence of complications post surgery significantly predict the post-surgery length of stay.

## Introduction

Degenerative cervical myelopathy (DCM), also known as cervical spondylotic myelopathy (CSM), is the most common cause of non-traumatic spinal cord dysfunction in the ageing population [1]. It is caused by the progressive narrowing of the spinal canal in the cervical region usually, but not solely, due to age-related degenerative changes. The timeline of this disease is characterised by either quiescent disease with episodes of symptom progression or continuous progressive deterioration. Furthermore, 20-62% of patients with DCM who do not undergo surgical treatment will deteriorate within three to six years [2]. Degenerative changes can occur at any cervical level, most commonly at the level of C5-C6 then C6-C7 and C4-C5 [3]. Degenerative changes include spondylosis, intervertebral disc disease, posterior longitudinal ligament hypertrophy, and ossification as well as congenital canal stenosis [4,5]. Cervical spinal nerves can be compressed as they exit the spinal cord and by the presence of accessory transverse foramen [6]. Important symptoms associated with DCM include hand paresthesia, loss of dexterity, issues with balance and coordination, muscle weakness, gait disturbances as well as the presence of Hoffman's sign and an inverted brachioradialis reflex [7].

The aim of this report is to discuss the findings of a retrospective study of the surgical management of DCM in Northern Ireland, a three-year retrospective study with a one-year follow-up. This study endeavours to contribute to the existing literature by providing insight into the surgical management of DCM in Northern Ireland. This study aims to uncover patterns in surgical management and outcomes for this population. Furthermore, by analysing the outcomes assessed by specific scoring systems at different time points of a patient’s journey, this study seeks to highlight the efficacy and potential areas of improvement in the management of DCM within Northern Ireland.

## Materials and methods

Study design

This is a retrospective study of prospective data collected from the Fracture Outcome Research Database (FORD). The FORD database takes into account patients presenting with all orthopaedic pathologies including non-traumatic pathologies, i.e. myelopathy, metastatic cord compression, nerve impingement, as well as fracture and dislocation. These patients selected were coded as having a diagnosis of DCM which was confirmed through clinical notes and imaging by the researchers in this study. Data were collected on a secure Excel sheet (Microsoft Software, Redmond, Washington, United States) on the hospital Citrix workspace (Citrix Systems, Inc.Fort Lauderdale, Florida, United States). A total of 102/104 consecutive patients (98.1%) with a diagnosis of degenerative cervical myelopathy, treated in Northern Ireland between 2019 and 2021 were evaluated. Two patients were removed due to incorrect coding of DCM diagnosis and were not included. The study was registered with the Audit and Quality Improvement Department in the Royal Victoria Hospital, Belfast. A formal ethics approval or Institutional Review Board approval was not required as per guidelines from National Health Service Health Research Authority (NHS HRA) regulations.

Inclusion and exclusion criteria

All patients diagnosed with degenerative cervical myelopathy, admitted to the Northern Ireland unit between 2019 and 2021, and treated either surgically or conservatively were included in this study. Exclusion criteria included all patients diagnosed with spinal tumour, fracture, central cord syndrome, and dislocation.

Data collection

All DCM patients coded in the FORD database were identified as per the inclusion criteria. Demographic data including gender, age, date of admission, date of discharge, post-surgery length of stay, place of residence, and co-morbidities were extracted from this database. The Northern Ireland Electronic Care Record (NIECR) was used to obtain admission and discharge letters, operation notes to identify the type of surgical management, inflammatory and infection markers post-operatively, and outpatient clinic letters to measure functional and urological outcomes via the modified Japanese Orthopaedic Association (mJOA) score and Medical Research Council (MRC) score [8]. The NIECR was also used to identify 30-day and one-year post-surgery mortality and morbidity. Imaging modalities used included Sectra IDS7 (Sectra AB, Linköping, Sweden), Uniview (Zhejiang Uniview Technologies Co., Ltd., Hangzhou, China), and picture archiving and communication system (PACS) to identify the level of DCM involvement and assess the severity of compression based on imaging and mJOA score.

Definitions and data variables

A diagnosis of DCM was confirmed based on coding in the FORD database, outpatient clinic notes diagnosing DCM based on symptoms, and reviewing imaging to confirm the level and severity of DCM. The choice of surgery was based on the maximal angle of compression, the number of vertebral levels involved, patient comorbidities, and anesthetic risk. Patients were stratified into age categories (<40, 40-60, and >60 years old) and gender (male and female) to compare outcomes. Co-morbidities were classified as musculoskeletal (Osteoarthritis, Osteopenia, Osteoporosis, Rheumatoid Arthritis, Seronegative Arthritis), renal (Chronic Kidney Disease, BPH, prostate cancer), endocrine (Diabetes Mellitus), cardiovascular (Heart failure, Aortic Stenosis). These co-morbidities were used to evaluate management options and the effect of co-morbidities on mJOA scores. All cervical vertebrae from C1 to C7 were assessed for signs of DCM and severity of stenosis using the three imaging modalities Sectra IDS7, Uniview, and PACs.The type of surgery included anterior cervical discectomy and fusion (ACDF), corpectomy, and posterior approach (laminectomy ± fusion). Preoperative symptoms were defined as upper and lower limb weakness, shooting/burning pain, and bowel/bladder incontinence. Patients were given a score out of three based on the number of symptoms present (1 = one symptom, 2 = two symptoms, 3 = more than two symptoms). Two scoring methods were used to assess functional and neurological outcomes. The mJOA score ranges from 0-18, 18 is no loss of function, mild (15-17), moderate (12-14), severe (0-11) DCM, and considers sensory and motor functional deficits and loss of bladder and bowel control. The MRC scoring system grades strength in the upper limbs and lower limbs out of 5 (0/5 = no power and 5/5 = normal power). Outpatient clinic appointments for follow-up were divided into four intervals: (i) pre-surgery, (ii) 48 hours post surgery, (iii) six months post surgery, and (iv) one year post surgery. The NIECR was reviewed to ascertain 30-day and one-year mortality and morbidity in all patients included in the study.

Data analysis

All data were collected by two researchers and analysed using IBM SPSS Statistics for Windows, Version 28.0 (Released 2022; Armonk, New York, United States) and Microsoft Excel 2022. A multiple linear regression test was used to analyse if the type of surgery, presence of complications, pre-surgery mJOA score, age, gender, and the presence of comorbidities significantly predicted the post-surgery length of stay. The rate of complications for each type of surgery was calculated using the following equation: number of complications for one type of surgery/total number of patients receiving that type of surgery X 100. The rate of wound infection for each surgery type was calculated using the following equation: number of wound infections for one type of surgery/total number of patients receiving that type of surgery X 100. A repeated measures ANOVA test was used to compare the effect of time (pre-surgery, 48 hrs, six months, and one year post surgery) on the average mJOA score. A one-way ANOVA test was conducted to compare the effect of age on the number of symptoms reported. A Wilcoxon signed-rank test was conducted to compare both MRC and mJOA scores between different time points. The R-value for each Wilcoxon signed-rank test was also generated to determine the size of the effect.

## Results

Patient demographics

**Figure 1:**
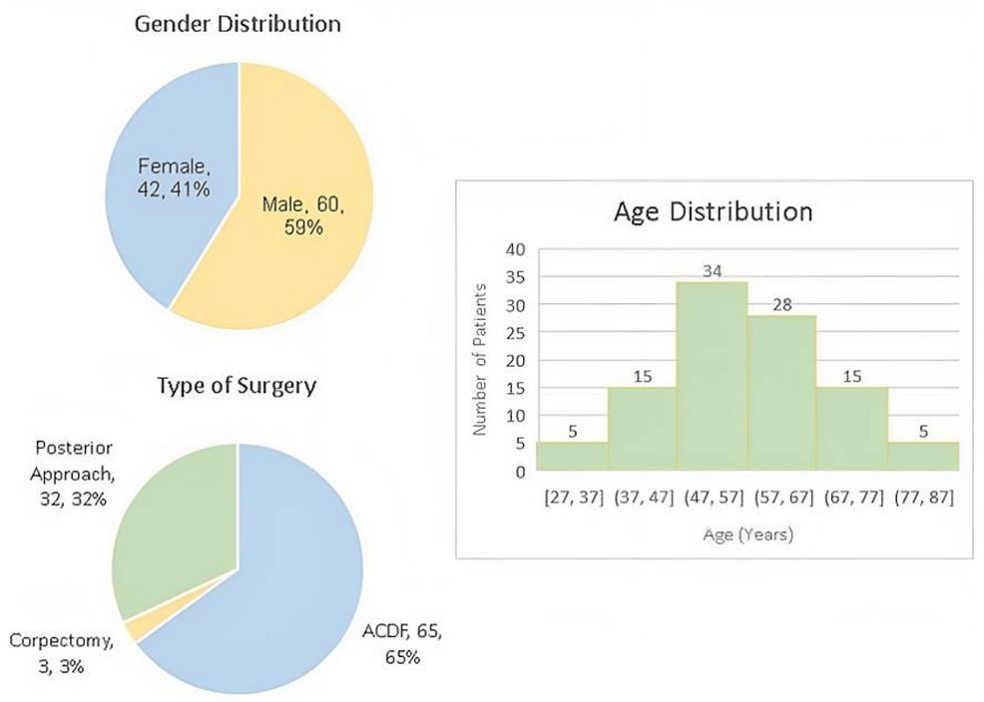
Demographics of the patient cohort. ACDF: Anterior Cervical Discectomy and Fusion

The sample consisted of 60 men (58.82%) and 42 women (41.17%) with an average age of 57.17 ± 12.13 years ranging from 27 to 83 years. Six patients had diabetes, seven had osteoarthritis, two had osteoporosis, and one had osteopenia. Sixty-seven patients underwent anterior surgery, 64 ACDF, and three corpectomy; 31 patients underwent posterior laminectomy with one patient receiving both an ACDF first and then a posterior laminectomy (Figure 1).

Effect of age, gender, comorbidities, type of surgery, post-surgery complications, pre-surgery mJOA score on post-surgery length of stay

Thirty-four patients had a length of post-surgery hospital stay over three days, with 21/34 (61.8%) patients having received a posterior approach surgery, 21 patients had a length of hospital stay post-surgery over seven days, with 15/21 (71.4%) patients having received a posterior approach surgery. The fitted regression model for the multiple linear regression test was: Type of Surgery = 26.165 - 3.438*(type of surgery) - 5.791*(complications post-surgery) - 1.044*(mJOA score pre-surgery) + 0.140*(age) - 0.232*(gender) - 1.496*(comorbidites). The overall regression was statistically significant (R2 = 0.223, F(6, 93) = 4.458, p = <0.001). Firstly, It was found that the pre-surgery mJOA score significantly predicted the length of stay post-surgery (β -1.044, p = 0.011). Secondly, it was found that post-surgery complications significantly predicted the post-surgery length of stay (β -5.791, p = 0.028). Both β coefficients for pre-surgery mJOA score and post-surgery complications are negative, which suggests that higher pre-surgery mJOA score and the presence of complications are associated with shorter lengths of stay. Thirdly, it was found that type of surgery did not significantly predict post-surgery length of stay (β -3.438, p = 0.060). The lack of conventional statistical significance may be influenced by the sample size and the specific number of patients within each type of surgery group. Two patients received a corpectomy and they had a length of post-surgery hospital stay of 25 days and one day, respectively. Three patients were treated conservatively due to patient choice, comorbidities, and poor surgical candidacy (Table 1). Seventy-one patients underwent surgery within six months of diagnosis while 28 patients underwent surgery at least six months to two years after diagnosis.

**Table 1:** Comorbidities and factors leading to conservative treatment for three patients. F: Female; M: Male; COVID-19: coronavirus disease 2019

Patient (Age/ Gender)	Level affected and severity	Comorbidities and reason for conservative treatment
1 (51, F)	C5-C6 Mild	Patient’s radicular symptoms resolved completely prior to operation and opted for conservative management.
2 (80, M)	C3-C4 Severe	Multiple comorbidities: heart failure, atrial fibrillation, obesity. Patient was deemed unfit and high risk for surgery due to poor physiological baseline.
3 (71, F)	C3-C5 Moderate	Unable to accommodate surgery due to COVID-19 and no beds available in the high-dependency unit post-operation. Currently on the waiting list.

Comparison of Post-Surgical Complications between Different Types of Surgery

The overall rate of complications was 13.7% with the highest rate of complications associated with corpectomy (33.3%) compared to posterior approach surgery (15.6%) and anterior cervical discectomy and fusion (ACDF) (12.3%). The most common complication was post-surgery wound infection (64.3%). The highest incidence rate of post-surgery wound infection occurred with posterior approach surgery (9.4%) compared to ACDF (7.7%). The types of complications can be seen in Table 2.

**Table 2:** Post-surgical complications associated with ACDF, corpectomy, and posterior surgery within the patient cohort. ACDF: Anterior Cervical Discectomy and Fusion

Complication	Number of patients	Surgery Type	Notes
Infection	9	6 ACDF, 3 Posterior	Two posterior cases resulted with infection involving multiple levels. One post-operative ACDF case developed urosepsis.
Failure of metalwork	1	Corpectomy	This resulted in reoperation by posterior stabilisation of C3 to T3.
Haematoma	1	Posterior	Required re-operation
Dysphagia – query laryngeal palsy	1	ACDF	
Urinary retention	1	ACDF	
Post-surgery oedema	1	Posterior	

Thirty days and One-year Mortality Rate Post-Surgery

Between 2019 and 2021, this patient cohort had a mortality rate of 0.98% (1/102). This patient died at one-year post-surgery aged 69 and had no comorbidities or complications. They received their surgery within six months of diagnosis and died of septic shock secondary to myelodysplastic syndrome.

Number of Patient Reported Symptoms Pre-Surgery and Bladder and Bowel Dysfunction

The number of patients reporting one symptom was 24/102 (23.5%), two symptoms were 69/102 (67.6%) and more than two symptoms were 9/102 (8.8%). The number of patients with bladder dysfunction pre-surgery was 15/102 (14.7%) and this improved by 1.9% to 13/102 (12.8%) patients 1-year post-surgery.

mJOA Scores at Four Different Time Intervals

**Figure 2:**
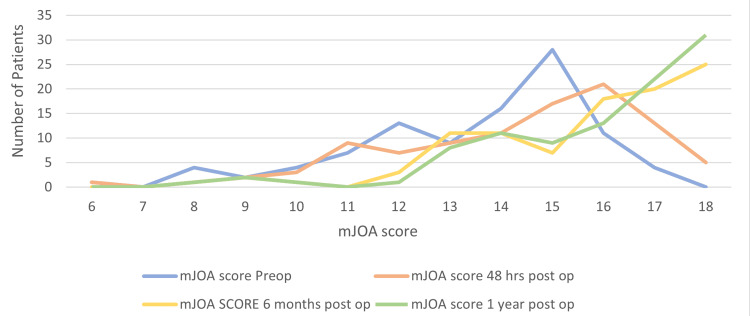
Distribution of mJOA scores at pre-surgery, 48 hours post surgery, six months post surgery, and one year post surgery. mJOA: modified Japanese Orthopaedic Association

The mJOA score was used to assess each patient’s motor function and sensation in the upper and lower limbs as well as their urinary function. The total score is out of 18 which indicates normal function within all subcategories. The average mJOA score pre-surgery was 13.44 ± 2.34. At 48 hours post-surgery it was 14.25 ± 2.52. At six months post surgery, it was 15.57 ± 2.4. At one year post surgery, it was 15.85 ± 2.37. The distribution of mJOA scores at each time point can be seen in Figure 2.

A repeated measures ANOVA was conducted to compare the effect of time (Pre-surgery, 48 hours, six months, and one year after surgery) on the mJOA score. There was a statistically significant difference in the mJOA score between at least two groups (Wilks’ Lambda = 0.26, F (3,96) = 91.095, p < 0.001) which suggests that mJOA score changes significantly from pre-surgery up to one year post surgery. This effect did not differ between the following age categories (<40, 40-60, and >60 years old), Wilk’s lambda =0 .96, F (6,188) p = .651, p = 0.689. However, each age category demonstrated improvement in mJOA score at each time point. A one-way ANOVA was conducted to compare the effect of age on the number of symptoms reported. This revealed that there was not a statistically significant difference in the number of symptoms reported between at least two groups (F (2,99) = 2.923, p = 0.058). Table 3 displays the results of statistical tests comparing different variables.

**Table 3:** Different statistical tests for the association between different variables. mJOA: modified Japanese Orthopaedic Association score

Predictor variable	Outcome variable	Significance test	Test result	p-value
Severity of stenosis on imaging	Surgery within 6 months of diagnosis	Chi-squared test	X^2 ^(2, N = 99) = 0.352	.839
Severity of stenosis based on pre-surgery mJOA score	Surgery within 6 months of diagnosis	Chi-squared test	X^2 ^(2, N = 99) = 1.546	.462
Surgery within six months of diagnosis	mJOA score at 1 year post surgery	Mann Whitney U test	Z = -1.536	.125
Gender	Number of symptoms	Chi-squared test	X^2 ^(2, N = 102) = 0.80	.67

The Results of Wilcoxon Signed Rank Test comparing MRC and mJOA scores between Different Time Intervals

Table 4 displays the results of a Wilcoxon signed-rank test performed to determine if there is a statistically significant difference in both MRC and mJOA scores between different time points (Pre-surgery, 48 hours, six months, and one year post surgery). A total of 99 patients were included in the analysis. If the R-value is more than 0.5, it suggests time has a large effect at different time points. The most notable effect size was the difference in mJOA score pre-surgery and one year post surgery (R = 0.579053, p<0.001). This test was also performed to determine if there is a statistically significant difference in MRC and mJOA scores for patients treated conservatively at diagnosis, 48 hours, six months, and one year after surgery. However, this was insufficient as there were fewer than five pairs in this group.

**Table 4:** Wilcoxon Signed Rank Tests on how the MRC and mJOA scores differ at different time points (pre-surgery and 48 hours, six months, and one year post surgery) MRC: Medical Research Council; mJOA: modified Japanese Orthopaedic Association

Variables	Median Values	Z-value	P-value	R-value
MRC score for Upper Limbs (Pre-Surgery and 6 Months Post-Surgery)	Pre-surgery 4.00, 6 months 5.00	-7.792	< 0.001	0.553753
MRC score for Upper Limbs (Pre-Surgery and 1 Year Post-Surgery)	Pre-surgery 4.00, 1 Year 5.00	-7.878	< 0.001	0.559865
MRC score for Upper Limbs (48 hrs and 1 Year Post-Surgery)	48 hrs 4.00, 1 Year 5.00	-6.887	< 0.001	0.489437
mJOA score (Pre-Surgery and 48 hours Post-Surgery)	Pre-surgery 14.00, 48 Hrs 15.00	-5.989	< 0.001	0.425619
mJOA score (Pre-Surgery and 6 months Post-Surgery)	Pre-surgery 14.00, 6 months 16.00	-8.108	< 0.001	0.576210
mJOA score (Pre-Surgery and 1 Year Post-Surgery)	Pre-surgery 14.00, 1 Year 17.00	-8.148	< 0.001	0.579053
mJOA score (48 hrs and 6 months Post-Surgery)	48 Hrs 15.00, 6 Months 16.00	-7.608	< 0.001	0.540677
mJOA score (48 hrs and 1 Year Post-Surgery)	48 Hrs 15.00, 1 Year 17,00	-7.889	< 0.001	0.560646

## Discussion

The first key finding of this study is that the mJOA score improves significantly post-surgery, particularly at six months and one year. The second key finding is that anterior approach surgery is associated with a lower rate of complications and shorter length of stay in hospital post-surgery compared to posterior approach surgery. The third key finding is that the mJOA score pre-surgery and the presence of complications post-surgery significantly predict the length of stay post-surgery. The type of surgery did not significantly predict the length of stay post-surgery however the p-value of 0.06 is close to the conventional statistically significant p-value of 0.05.

Patient demographics

The results of the current study agree with the existing literature that DCM primarily affects those who are male and in their 50s and 60s [9,10].

Comparison of post-surgical complications between different types of surgery

The most common surgical approach in this study was ACDF. This is significant as ACDF had the lowest rate of complications in this study compared to corpectomy and a posterior approach surgery [11]. Post-surgery wound infection was highlighted as the most common complication, particularly with posterior approach surgery. This is significant for clinicians to be aware of as they may want to consider prophylactic antibiotics or be extra cautious in observing for clinical signs of infection. A retrospective cohort study by Nunna et al. [12] also found a higher rate of post-operative complications associated with a posterior approach compared to an anterior approach. A posterior approach was also associated with a higher rate of wound infection. The study by Nunna et al. [12] relied solely on ICD codes which may result in underreporting or inaccurate codes. These may have been logged incorrectly, for example, as central cord syndrome. One patient in this cohort reported dysphagia after an ACDF which also correlates with the literature [12].

No patients in this review reported a C5 palsy complication. A C5 palsy is more common with posterior approach surgery due to the short C5 nerve root as it exits the spinal cord and if multiple cervical levels are involved [13]. A large database study of 102,807 patients found a statistically significant higher incidence rate of C5 palsy with posterior laminectomy and fusion (4.84%) compared to ACDF (1.74%) [11]. The lack of C5 palsy complications reported in the current study may be due to the favoured ACDF approach, patient surgical profiles, or lack of documentation.

Role of type of surgery, post-surgery complications, pre-surgery mJOA score, age, gender and comorbidities on predicting the post-surgery length of stay

The current study found that patients receiving posterior approach surgery had a longer length of stay after surgery compared to patients who received an ACDF. This could be because these patients had multiple cervical levels involved, some patients had severe DCM and there was a higher rate of complications associated with a posterior surgical approach. They may have experienced more postoperative pain due to the posterior cervical spinal anatomy that is affected [12,13]. This is significant for healthcare institutions involved in surgical planning as the care for these patients could be planned ahead of time if it is likely that they will need a hospital bed for an extended length of time post-surgery. The decision of surgical approach should be made with each specific patient in mind. The choice will depend on the level or levels of compression, the presence of comorbidities, imaging factors, and the joint decision of the clinician and patient. When comparing the effect of the type of surgery on predicting the length of stay post-surgery within the multiple linear regression test the association was not statistically significant, however, the p-value was 0.06 which is very close.

Time to surgery and patient outcomes

A study by Hilton et al. states that surgery within six months of symptom onset offers the best chance to minimise disability and allow for recovery [14]. This study was conducted in a single centre and only included patient imaging taken from that centre. This may have led to a biased result which may not provide an accurate picture of the entire population. This study, however, did not explore reasons for the delay of surgical intervention. The current study found no significant difference in mJOA score at one year post surgery between patients who received surgery within six months of diagnosis and those who did not. This would suggest that the time to surgery may not be a key factor for a patient’s outcome as this disease is often slow to progress. However, despite this finding, timely diagnosis and prompt surgical intervention are vital for patients as the speed of progression of DCM remains unknown.

Thirty-day and one-year post-surgery mortality rate

The current study found that surgical management for DCM in Northern Ireland has an extremely low mortality rate of 0.98% by one year post surgery. This is reassuring that the provision of this service is safe and effective. This patient received surgery within six months and so time to surgery was not considered a factor in their outcome. An observational study by Davies et al. reported a mortality rate of 2.29% within two years post surgery [15]. Davies et al. found that older age at operation and severe DCM resulted in worse survival [15]. Interestingly, this study highlights that surgical intervention in the early stage of disease may positively impact life expectancy. A limitation of this study is the absence of specific causes of death for the patients included. It is difficult to conclude if these patients died specifically due to the presence of DCM or other comorbidities. Furthermore, the patient data was collected from 1994 to 2007 and may not be representative of the population and treatment options available today.

Number of patient-reported pre-surgery symptoms and bladder and bowel dysfunction

A high percentage of patients reported two symptoms at the time of diagnosis. This was either motor, sensory, or bladder dysfunction in keeping with symptoms of DCM. The small improvement in bladder dysfunction for a small number of patients is also in accordance with the literature as few patients regain this control after surgery [3]. A prospective study of 25 patients reported only one out of seven patients (14.2%) with improved sphincter dysfunction post surgery [16]. The prospective nature and accurate data collection is a strength of this study; however, the sample size is very small. Additional studies such as a prospective multicentre study with larger sample sizes would provide more robust results.

mJOA scores at four different time intervals

A key part of the current study identified that the mJOA score significantly improved after surgery, particularly by six months and one year. This finding is reassuring that the provision of this service within Northern Ireland is valuable to this patient population and provides evidence that this service is useful and beneficial. An improvement in mJOA score by six months is significant for clinicians as they can use this to guide patient expectations and consider when to review them post-surgery. The mJOA score can be used to assess patient improvement pre- and post-surgery. Age was not found to be a significant factor in mJOA score improvement. This means that while younger patients often had better pre-surgery mJOA scores, their improvement post-surgery was no better than those with advanced age. However, the under 40 category had only seven patients, while the 40-60 category had 55 patients. This makes it difficult to draw accurate conclusions about age from this dataset. A retrospective chart audit of 460 patients by Zhang et al. also found that patients had their greatest rate of recovery within six months after surgery [17]. They found that patients within each age category had functional improvement after surgery. This study had a moderately large sample size, included data pre- and post-surgery as well as imaging, and considered comorbidities when completing statistical analysis. A few limitations of this study by Zhang et al. include its retrospective design and single-centre study, which could introduce selection bias and limit the application of these findings to other populations [17]. The follow-up period of six months was quite short and may not capture long-term outcomes.

Strengths

The current study has several strengths that support the findings and recommendations. This study had a large consecutive patient cohort from a regional institution that covers all of Northern Ireland completed over three years. Each patient within the cohort had a complete one-year follow-up and patient notes were recorded in great detail by clinicians. The current study was able to compare outcomes over time which is useful to assess the trends in patient recovery and improvement post-surgery.

Limitations

Due to the retrospective nature of this study, there may be limitations regarding the data extracted from the database. Despite the detailed documentation by clinicians, there may be information bias associated with the retrospective evaluation of the data. A few patients had comorbidities that may have affected their mJOA assessment and score. Lastly, this study did not explore the specific mechanism of cord compression that resulted in DCM and did not include patient-reported outcomes that could have been assessed by the Short Form - 36 (SF-36) and Neck Disability Index (NDI).

Future directions

Based on the findings of this study, we would suggest a further prospective multi-centre study. This would increase the impact of these findings and allow a comparison of outcomes among different centres and populations. We would also ask our clinicians to include specific documentation of mJOA scores at each time point. This could be included in the existing clerk in proformas used by clinicians. This would also include a longer follow-up of two years with patient-reported outcomes in the form of quality-of-life scores such as the SF-36 and NDI.

## Conclusions

Overall, this retrospective study is beneficial as it evaluates information about a patient population from a regional spinal centre and reflects the findings of the current literature. The first key finding is that the mJOA score improves significantly post-surgery, particularly at six months and one year post surgery for the majority of patients. The second key finding is that anterior approach surgery is associated with a lower rate of complications and shorter length of stay in hospital post-surgery compared to posterior approach surgery. Furthermore, this study has found that the mJOA score pre-surgery and the presence of complications post-surgery significantly predict the length of stay post-surgery. These results can help clinicians plan aspects of post-surgery care based on a patient's pre-surgery mJOA score and associated complications with the type of surgery involved.
